# Exploring Use of Digital Health Technologies, Digital Health Care Literacy, and Attitudes Toward Digital Health Among Norwegian Health Care Personnel Involved in Home-Based Pediatric Palliative Care: Cross-Sectional Study

**DOI:** 10.2196/74928

**Published:** 2026-02-26

**Authors:** Judith Schröder, Kirsti Riiser, Heidi Holmen

**Affiliations:** 1 Department of Nursing and Health Promotion, Faculty of Health Sciences, OsloMet – Oslo Metropolitan University, P.O. Box 4, St. Olavs plass, Oslo, 0130, Norway, 4794841823; 2 Department of Rehabilitation Science and Health Technology, Faculty of Health Sciences, OsloMet – Oslo Metropolitan University, Oslo, Norway

**Keywords:** digital health technologies, digital health, eHealth, palliative care for children, health care personnel, home-based, health care literacy, attitudes, home

## Abstract

**Background:**

Digital health technologies can potentially increase the efficiency and quality of pediatric palliative care (PPC), yet their use in home-based PPC remains limited. Limited digital health care literacy and inadequate training can reduce confidence and foster negative attitudes, whereas positive experiences and basic digital health care literacy may encourage adoption.

**Objective:**

This study aims to explore the use of digital health technologies by Norwegian health care personnel in home-based PPC and examine the association between their digital health care literacy and their attitudes toward digital health.

**Methods:**

A cross-sectional study was conducted from September 2023 to May 2024, with an online survey targeting health care personnel involved in home-based PPC through primary or specialist health care services. Data were collected using selected items from the Norwegian Healthcare Personnel Survey on eHealth 2022, the Digital Health Care Literacy Scale (DHLS), and the Information Technology Attitude Scales for Health (ITASH), alongside demographic characteristics. Higher DHLS scores indicate greater digital health care literacy, while higher ITASH scores reflect more positive attitudes toward digital health technologies. Pearson correlation, ANOVA, and multiple linear regression analyses were conducted to comprehensively explore the relationships and associations among the variables.

**Results:**

Health care personnel (n=148) from diverse health care services responded to the survey. Half of the respondents (72/144, 50%) had experience with real-time video consultation, while phone calls were the primary communication method (138/145, 95.2%). Additionally, 55.6% (79/142) of the respondents had limited or minimal access to electronic health records from other health care services. Health care personnel perceived digital health technologies for remote PPC as a supplement (126/135, 93.3%) rather than a replacement for in-person care. Mean digital health care literacy was 18.29 (SD 3.8) on a scale from 0 to 23. On a scale from 1 to 4, the highest recorded scores pertained to attitudes toward digital health technologies in supporting care (mean 3.17, SD 0.39) and the perceived need for training (mean 3.16, SD 0.43). A statistically significant association was found between the respondents’ level of digital health care literacy and their attitudes toward digital health technologies in supporting care (β=0.030, 95% CI 0.014‐0.047*; P*<.001).

**Conclusions:**

This study examined the use of digital health technologies by Norwegian health care personnel in home-based PPC, their digital health care literacy, and attitudes toward digital health. Despite positive attitudes and high digital health care literacy, use of digital health technologies was limited, suggesting that inadequate digital health solutions may hinder effective implementation. Addressing these barriers is crucial to enhancing the implementation of digital health in home-based PPC. Future research should focus on integrating digital health technologies into existing infrastructure and workflows while exploring their impact on personalized care to ensure high-quality home-based PPC.

## Introduction

Health care personnel play a crucial role in providing home-based pediatric palliative care (PPC) to children with life-threatening and life-limiting conditions. Their involvement is essential for implementing effective strategies and care tools that address the physical, psychological, and spiritual needs of these children and their families, thereby enhancing the quality of life of both the children and their families [1]. However, providing PPC at home presents unique challenges compared to hospital-based PPC, including limited availability of specialized personnel and the need for effective coordination among health care services [2,3]. Digital health has been suggested as a potential approach to support care delivery in this context, offering opportunities to improve communication, coordination, and continuity of care [2-7].

According to the World Health Organization, digital health refers to knowledge and practice associated with the development and use of digital health technologies to improve health*,* encompassing eHealth and emerging areas such as the use of advanced computing sciences in big data, artificial intelligence, and robotics [8]. In this cross-sectional study, digital health is used to denote this broader concept, while digital health technologies refer specifically to applications of information and communication technologies, such as eHealth records, telephone, and videoconferencing, that support health care personnel in providing home-based PPC.

Digital health can help mitigate limitations in the availability and expertise of health care personnel providing home-based PPC by supporting communication, documentation, remote monitoring, collaboration, or training [4-7,9,10]. For example, real-time video consultation enables families to receive guidance from PPC specialists without traveling [4-7,10,11] while access to electronic health records supports targeted care planning [10,12-14]. Despite these benefits, challenges such as technical failures, poor connectivity, lack of interoperability, and concerns about data privacy limit effective use [4-6,14]. Moreover, health care personnel often perceive that technology cannot replace the therapeutic value of in-person care [4-7,14]. Consequently, the use of digital health technologies to support care in home-based PPC remains limited [4-7].

Health care personnel’s digital health care literacy and attitudes toward digital health are critical for successful implementation [4,6,7]. Digital health care literacy refers to the ability to seek, understand, and use digital health technologies [15], while attitudes toward digital health technologies encompass perceptions and beliefs regarding their use in practice [16]. Limited digital health care literacy and inadequate training can reduce confidence and foster negative attitudes, whereas positive experiences and basic digital health care literacy may encourage adoption [16,17]. Understanding whether digital health care literacy is associated with attitudes toward technology could inform strategies to strengthen competence and increase technology use in home-based PPC.

In Norway, home-based PPC is publicly funded and provided by both primary and specialist health care services [18]. Approximately 8162 Norwegian children aged 0‐19 years may require palliative care, based on estimates adapted from England [19], with 20% living with cancer, 40% with congenital genetic conditions, and 40% with neuromuscular diseases [20]. National PPC guidelines published in 2016 provide a framework for best practices, emphasizing shared responsibility between hospitals and municipalities, structured collaboration, respite care, and multidisciplinary teams [21]. Despite these recommendations, there is currently no national overview of how PPC is implemented or which health care personnel provide these services [19].

Parallel to these efforts, Norway’s digital strategy and welfare technology program aim to integrate digital health into health care, including services for children [22]. In the Norwegian context, welfare technology typically encompasses digital tools and assistive technologies such as remote monitoring systems, communication platforms, safety alarms, and other digital health technologies that facilitate home-based care and reduce reliance on hospital services [22]. Successful integration requires time, resources, and collaboration among stakeholders, as emphasized in a Norwegian study [22]. Furthermore, improving the use of digital health technologies depends not only on the technology itself but also on the digital health care literacy of its users. According to the Digital Agenda for Norway, health care personnel with strong digital health care literacy are crucial for realizing the full benefits of digital health [23].

Norway faces a critical need to strengthen evidence on health care services for children requiring palliative care at home [19]. Although the national digital health strategy promotes technological integration, its potential remains largely unexplored in home-based PPC. Limitations persist in understanding health care personnel’s use of digital health technologies, their digital health care literacy, and their attitudes toward digital health. Addressing these limitations is essential to ensure that digital health translates into meaningful improvements in home-based PPC. This cross-sectional study addresses these limitations by exploring current practices and perceptions, focusing on the use of digital health technologies in home-based PPC and the relationship between digital health care literacy and attitudes toward digital health. Results can inform Norwegian policy and contribute to the global discourse on digital health for home-based PPC and may also offer transferable strategies for improving home-based care supported by digital health technologies for other patient groups.

## Methods

### Study Design

An exploratory cross-sectional nationwide study was conducted using an online survey with a convenience sample to explore the use of digital health technologies, digital health care literacy, and attitudes toward digital health among health care personnel providing home-based PPC in diverse health care services in Norway. The online survey was launched on September 14, 2023, and remained open until May 3, 2024.

Reporting adheres to the STROBE (Strengthening the Reporting of Observational Studies in Epidemiology) [24,25] and the CHERRIES (Checklist for Reporting Results of Internet E-Surveys) guidelines [26].

### Participants and Data Collection

The participants eligible for this study were medical, nursing, and allied health care personnel, including physiotherapists, occupational therapists, social workers, or psychologists, who were currently or previously involved in home-based PPC through primary (community-based) or specialist health care services (hospital-based).

The first author (JS) sourced the email addresses of health care personnel from the official websites of all, at the time, 356 Norwegian municipalities and sent the survey link with information about the study and an invitation to participate via email. The survey link was also distributed through the research network Children in Palliative Care (CHIP) [27], which the CHIP homeTec project [28] and, consequently, this study, is a part of. Additional recruitment was carried out via the official CHIP HomeTec Instagram account, the CHIP HomeTec blog, and newsletters distributed by national health care personnel associations.

### The Online Survey and Measurements

The survey included selected items of the Norwegian Healthcare Personnel Survey on eHealth 2022 [29], the Digital Health Care Literacy Scale (DHLS) [15], the short version of the Information Technology Attitude Scales for Health (ITASH) [30,31], and demographic items, as shown in Table 1. Each section of the survey was displayed on a separate page in the digital survey tool.

**Table 1.** Overview of measures in the survey.

Scale and content	Items	Response scale
Norwegian Healthcare Personnel Survey on eHealth 2022		
Usage of digital health technologies when providing health care services	4	Multiple choice questions with a single or multiple responses
Perceptions toward digital health care services	11	Six-point Likert scale: 1=strongly disagree to 5=strongly agree, and 0=not relevant Five-point Likert scale: 1=strongly disagree to 4=strongly agree, and 0=not relevant
Information Technology Attitude Scales for Health		
Subscale: care value of technology	4	Four-point Likert scale: 1=strongly disagree to 4=strongly agree
Subscale: training in technology skills	6	Four-point Likert scale: 1=strongly disagree to 4=strongly agree
Subscale: confidence in technology	6	Four-point Likert scale: 1=strongly disagree to 4=strongly agree
Subscale: technology-related workload	5	Four-point Likert scale: 1=strongly disagree to 4=strongly agree
Digital Health Care Literacy Scale^a^		
Items about installing apps/programs, using apps/programs, setting up video calls, and solving minor technical problems without relying on external assistance	4	Five-point Likert scale: 1=strongly disagree to 5=strongly agree
One item about action when encountering a technical problem	1	1=ask someone for help and 2=try to solve the technical problem on my own – without help
Frequency of needing help with digital devices	1	Five-point Likert scale: 1=always to 5=never
Demographic items		
Age, gender, profession, additional education, management experiences, workplace, geographical work location, and county of work	12	Multiple-choice questions with a single response and 2 open-ended questions

a Sum score of six items (range 6-23). A higher score reflects a higher level of digital health care literacy.

### The Norwegian Healthcare Personnel Survey on eHealth

To explore the digital health technology use and perceptions, the Norwegian Healthcare Personnel Survey on eHealth 2022, assessing health care personnel’s usage, perceptions, and satisfaction regarding digital health care services, was reviewed [29]. The survey comprised 29 items; of these, 15 were deemed relevant and included in this cross-sectional study, while 14 were excluded to avoid redundancy with the ITASH and DHLS scales (Table 1). The items examine how frequently and in what ways digital health technologies are used by the participants, as well as their perceptions of digital health care services. The included items used 6-point and 5-point Likert scales and multiple-choice responses. The Norwegian Healthcare Personnel Survey on eHealth was developed for the annual review of eHealth in the Norwegian health care services starting in 2019, but has not been validated.

### ITASH

The short version of the ITASH was included to assess attitudes toward information and communication technology [30]. The instrument is translated and validated in Norwegian [32]. Permission for use was obtained from the developer [31,32]. ITASH comprises 21 items organized into 4 subscales that evaluate attitudes toward the care value of technology (4 items), training in technology skills and the desire for additional training (6 items), confidence in dealing with technology (6 items), and technology-related workload (5 items) (Table 1). All items are rated on a 4-point Likert scale, ranging from 1 = “strongly disagree” to 4 = “strongly agree.” Each subscale’s mean score is calculated and ranked, with higher scores on a scale from 1 to 4 indicating more positive attitudes toward digital health technologies. In this study, the Cronbach α for each subscale was 0.78 (care value), 0.77 (training), 0.87 (confidence), and 0.79 (technology-related workload), indicating acceptable internal consistency for the ITASH subscales.

### Digital Health Care Literacy Scale

The Digital Health Care Literacy (DHLS) scale was developed and validated by Nelson et al [15] to assess the foundational digital health care literacy of caregivers of children receiving care at a pediatric clinic. The items were designed to be theoretically applicable to a wide range of individuals [15]. Foundational digital health care literacy encompasses the basic abilities and knowledge necessary to confidently and efficiently use digital technologies and navigate the digital platforms used in health care [15]. With permission from the developers of DHLS, the CHIP homeTec research group translated the scale from English into Norwegian using a forward-backward method [33]. To explore the relevance of the DHLS scale in a Norwegian context, all 6 items were included. Although previous research indicates a model with 3 items may be sufficient [15], we chose to retain all 6 items because this was the first time the scale was applied in Norway, and a comprehensive approach was necessary to assess its applicability in a later, separate evaluation. The 6 items (Table 1) evaluate confidence in using technological programs or services, as well as the ability to troubleshoot technical issues [15]. Following the syntax for DHLS scoring, responses to item 5 were recoded from 0 to 1 and 1 to 3, to align with other variables from this scale before calculating the total sum score [15]. The DHLS items were scored by summing all 6 items, resulting in a total DHLS sum score ranging from 0 to 23 [15]. A higher DHLS sum score indicates a higher level of digital health care literacy. In this study, the Cronbach α reliability coefficient for the DHLS scale was 0.86.

### Demographic Characteristics

Demographic characteristics included age, gender, profession, additional education, management experiences, workplace, geographical work location, and county of work (Table 1). Participants were asked to report their age because it may influence health care personnel’s attitudes toward digital health technologies due to differences in technological familiarity and comfort levels [34]. Workplace (categorized as either primary or specialized health care services) and geographical work location (determined by the associated driving distance) are considered organizational factors that may influence the attitudes of health care personnel [7,35,36]. Lastly, we included a variable for prior use of real-time video consultation, which participants reported as either “yes” or “no,” with “yes” denoting previous experience that may influence their attitudes toward digital health technologies [37].

To simplify the presentation and analysis of the participants’ characteristics, some variables were recoded. The variables nurse (36/148, 24.3%), public health nurse (6/148, 4%), and social educator (6/148, 4%) were combined under the term nurse due to their similar responsibilities within nursing, including clinical care, public health, and health education. The variable county was collapsed into the 4 regional health authorities, which are widely recognized throughout Norway, reducing the number of categories from 11 counties (the number of counties in Norway as of 2023) to 4 categories.

Furthermore, the participants’ workplaces were recategorized into the dichotomous variables of primary care and specialist care. General practitioners (3/148, 2%) were included in the primary care category, while private health care services (2/148, 1.35%) and others (3/148, 2%) were excluded from the bivariate Pearson correlation (*r*) analysis due to the small sample sizes, which would not provide reliable statistical power for the analysis [38].

The survey underwent pilot testing by the CHIP homeTec network members, comprising professionals from diverse disciplines [28]. This pilot phase aimed to gather reflections on the relevance, comprehensiveness, and overall balance of the items used [38].

### Statistical Analysis

Missing values were not imputed due to the exploratory nature of the study, and incomplete questionnaires (participants responding to less than 80% of the items) were excluded (2/150, 1.3%) [39]. All other responses were included using SPSS’s pairwise deletion function, which excludes cases only when they have missing values for the specific analysis being conducted [39]. Multiple entries to the survey were not a concern in this study, and no responses were withdrawn.

Categorical data are reported as frequencies (percentages), while continuous data are reported as mean (SD) if normally distributed and as median (IQR) if skewed. Pearson *r* was applied to examine the associations between the DHLS sum score and the variables of age, workplace, and prior use of real-time video consultation, as well as between the ITASH subscales and the same variables [39]. An analysis of variance (ANOVA) was used to determine whether there were statistically significant differences between the DHLS sum score and attitudes toward digital health technologies (ITASH subscales) among health care personnel based on the variable of work location, with associated driving distances. Bivariate statistics were used to explore the relationships between pairs of variables.

Both unadjusted and adjusted linear regression analyses were conducted to explore the association of the DHLS sum score with the 4 ITASH subscales, with the adjusted analysis accounting for demographic characteristics. The results are presented with coefficients (β), 95% CI, and *P* values. Statistical significance was set at *P*≤.05 for 2-tailed comparisons. The data were analyzed using the Statistical Package for the Social Sciences (SPSS), version 29.

### Ethical Considerations

This study was reviewed by the Norwegian Agency for Shared Services in Education and Research (reference number 657413) to ensure that well-considered information was provided to the participants and that the processing of personal data complied with data protection regulations and maintained the privacy of the participants. Consent forms and survey responses were separately stored in 2 distinct files to guarantee data deidentification and to uphold participant anonymity during the data analysis. Data collection was handled by the Nettskjema tool, a survey solution developed and hosted by the University of Oslo [40]. The data were subsequently stored in the Services for Sensitive Data system, which is specifically designed to handle and safeguard sensitive information following data protection regulations [41]. The initial page of the survey provided participants with accessible information about the research project, encompassing its objectives and highlighting their voluntary participation and the endeavors taken to secure the project data. Participants were given information about the study and required to provide digital consent via a Norwegian ID on the second page before proceeding to the survey on the third page.

## Results

### Sample Characteristics and the Use of Digital Health Technologies

Most respondents were female (140/148, 94.6%), with ages ranging from 25 to 67 years and a median age of 45 (37-52) years. Diverse professions were represented, with the largest group being physiotherapists (56/148, 37.8%), followed by nurses (48/148, 32.4%). The vast majority worked in primary care (117/144, 81.3%). The geographical distribution of respondents was 36.4% (51/140) from small towns with moderate driving distances, 34.3% (48/140) from rural areas with long driving distances, and 29.3% (41/140) from towns with walking distances or short drives Table 2.

**Table 2.** Demographic variables and reported use of digital health technologies.

Variables	Values
Age in years (n=142)^a^, median (IQR)	45 (37-52)
Sex (n=147)^a^, n (%)	
Female	140 (94.6)
Profession (N=148), n (%)	
Physiotherapist	56 (37.8)
Nurse^b^	48 (32.4)
Occupational therapist	23 (15.5)
Physician	11 (7.4)
Other	10 (6.8)
Workplace^c^ (n=144)^a^, n (%)	
Primary care^d^	117 (81.3)
Work location with associated driving distance (n=140)^a^, n (%)	
Small town, moderate driving distance	51 (36.4)
Rural, long driving distance	48 (34.3)
Town, walking distance or short drive	41 (29.3)
Regional Health Authorities Norway (N=148), n (%)	
South-Eastern	82 (55.4)
Western	27 (18.2)
Central	22 (14.9)
Northern	17 (11.5)
Reported use of digital health technologies (n=144)^a^, n (%)	
Prior use of real-time video consultation (yes)	72 (50)
Primary communication tools with other care providers, n (%)	
Phone: very often or sometimes (n=145)^a^	138 (95.2)
Digital messaging: very often or sometimes (n=146)^a^	125 (85.6)
Real-time video consultation: very often or sometimes (n=143)^a^	61 (42.7)
Digital access to patients’ electronic health records from other health care services (n=142)^a^, n (%)	
Very small extent or small extent	79 (55.6)
Neither large nor small extent	31 (21.8)
Very large extent or large extent	32 (22.5)
Digital health care services with patients are in addition to in-person care (n=135)^a^, n (%)	
Strongly agree or agree	126 (93.3)
Could your most recent physical patient contact have been replaced if available? (N=148), n (%)	
With real-time video consultation (yes)	29 (19.6)
With phone (yes)	13 (8.8)
With digital messaging (yes)	9 (6.1)
Require more options for digital health care services with patients (yes) (n=127)^a^, n (%)	96 (75.6)
Require more options for digital communication or digital health services with other care providers (yes) (n=135)^a^, n (%)	120 (88.9)

a Totals may not sum to 148 due to item non-response

b Includes public nurses (6/49) and social educators (6/49).

c Primary care or specialized care.

d Includes general practitioners (3/117).

Half of the respondents (72/144, 50%) reported prior experience with real-time video consultation. Phone calls were the most frequently used modality to communicate with other health care personnel who were engaged in the care of the child and family (138/145, 95.2%), followed by digital messaging (125/146, 85.6%). About half of the respondents reported having limited or minimal access to patients’ electronic health records from other health care services (79/142, 55.6%). Most respondents (126/135, 93.3%) agreed that digital health care services should be supplementary to in-person care. Only a minority reported that their most recent physical patient contact could have been replaced with real-time video consultation (29/148, 19.6%), phone calls (13/148, 8.8%), or digital messaging (9/148, 6.1%) if available. Furthermore, a large proportion of the respondents required more options for digital communication and digital health care services, both with patients (96/127, 75.6%) and other health care personnel (120/135, 88.9%; Table 2).

### Digital Health Care Literacy and Attitudes Toward Digital Health Technologies

The total sample DHLS mean score was 18.29 (SD 3.8). The ITASH subscale care value of technology displayed the highest mean score (mean 3.17, SD 0.39), followed by training in technology skills and the desire for additional training (mean 3.16, SD 0.43), confidence in dealing with technology (mean 2.55, SD 0.52), and reported technology-related workload (mean 1.95, SD 0.42) (Table 3).

The analysis showed a statistically significant negative correlation between the DHLS sum score and age (*r*=−0.485; *P*<.001). Additionally, there was a statistically significant negative correlation between ITASH confidence and workplace (*r*=−0.221; *P*=.008; Table 3). Other correlations were not statistically significant.

**Table 3.** Bivariate statistics to explore the correlation between the Digital Health Care Literacy Scale, Information Technology Attitude Scales for Health subscales, and demographic data.

Variables	DHLS^a^^,^^b^ score	ITASH^c^^,^^d^ Care value	ITASH Training	ITASH Confidence	ITASH Technology-related workload
Total sample, mean (SD), n	18.29 (3.8), 142	3.17 (0.39), 146	3.16 (0.43), 147	2.55 (0.52), 146	1.95 (0.42), 144
Age					
Participants, n	137	140	141	141	138
*r^e^*	–*.485*^f^	–.129	–.004	.031	.149
*P* value	<.001	.13	.96	.72	.08
Workplace					
Participants, n	138	142	143	142	140
*r^e^*	–.026	–.030	.153	–*.221*^f^	.010
*P* value	.76	.72	.07	.008	.90
Real-time video consultation					
Participants, n	138	142	143	142	140
r^e^	–.106	–.038	.030	–.008	.156
*P* value	.21	.65	.72	.92	.07
Work location with driving distance, mean (SD), n					
Small town, moderate driving distance	18.29 (4.19), 48	3.25 (0.43), 51	3.18 (0.41), 51	2.58 (0.30), 51	1.90 (0.40), 51
Rural, long driving distance	18.02 (3.93), 47	3.11 (0.39), 47	3.17 (0.47), 47	2.51 (0.18), 48	1.90 (0.45), 45
Town, walking distance or short drive	18.97 (3.62), 39	3.17 (0.33), 40	3.17 (0.43), 41	2.55 (0.25), 39	2.05 (0.42), 40
*P* value between groups^g^	.52	.19	1	.45	.20

a DHLS: Digital Health Care Literacy Scale.

b DHLS score: sum score of 6 items (min-max: 6‐23). Higher scores reflect a higher level of digital health care literacy.

c ITASH: Information Technology Attitude Scales for Health.

d ITASH: 4-point Likert scale 1=strongly disagree to 4=strongly agree.

e Pearson *r*.

f The correlation is significant at a significance level of ≤.05 (2-tailed).

g *P* values calculated by ANOVA.

The DHLS score was significantly associated with the ITASH subscale care value, in both the unadjusted (β=0.030, 95% CI 0.014-0.047) and adjusted (β=0.034, 95% CI 0.014-0.054) analyses (Table 4). This suggests that, when accounting for other factors, each one-point increase in the DHLS score is associated with an average increase of 0.034 points in the ITASH subscale care value score. The associations between the DHLS score and the ITASH subscales of training, confidence, and technology-related workload were not statistically significant.

**Table 4.** Digital Health Care Literacy Scale sum score associated with attitudes toward digital health technologies (Information Technology Attitude Scales for Health subscales).

	DHLS score			
	ITASH^a^ Care value^b^	ITASH Training^b^	ITASH Confidence^b^	ITASH Technology-related workload^b^
	Unstandardized B (95% CI)	Unstandardized B (95% CI)	Unstandardized B (95% CI)	Unstandardized B (95% CI)
Unadjusted	.030^c^ (0.014 to 0.047)	.001 (–0.018 to 0.019)	–.002 (–0.013 to 0.009)	–.017 (–0.035 to 0.002)
*P* value	<.001	.92	.70	.07
Adjusted^b^	.034^c^ (0.014 to 0.054)	.005 (–0.017 to 0.027)	–.008 (–0.020 to 0.003)	–.011 (–0.033 to 0.011)
*P* value	<.001	.68	.16	.33

a ITASH: Information Technology Attitude Scales for Health.

b Adjusted linear regression analysis with control for age, prior use of real-time video consultation, work location with associated driving distance, and workplace.

c Statistical significance at .05 (2-tailed).

## Discussion

### Principal Results

This study describes the use of digital health technologies, digital health care literacy, and attitudes toward digital health among health care personnel in diverse home-based PPC services. Half of the respondents had experience with real-time video consultation, while phone calls and digital messaging were the primary communication methods in and between health care services. More than half of the respondents had limited or minimal access to electronic health records from other health care services involved in the child’s care. The results also indicate that health care personnel perceive digital health technologies for remote home-based PPC as a supplement rather than a replacement for in-person care. However, the health care personnel in this study warrant more options for digital health to enable and support their interaction with children, families, and other care providers. The sample showed high digital health care literacy and positive attitudes toward digital health technologies. A statistically significant but weak association was identified between digital health care literacy and positive attitudes toward digital health technologies in supporting home-based PPC.

### Interpretation

Although the measured association in this study was weak and the direction of the association remains unclear due to the cross-sectional design [38], both digital health care literacy and attitudes toward digital health technologies appear to be important factors in the use of digital health technologies [10,17,37]. Even if digital health care literacy does not strongly or directly influence attitudes, its combined role in shaping health care personnel’s ability and willingness to use digital health technologies should not be ignored. A high level of digital health care literacy may enable health care personnel to use digital health technologies more effectively, while positive attitudes toward digital health may drive engagement and openness to learning new digital skills [17,37,42]. This underscores the need for targeted activities to support both digital health care literacy development and positive attitudes toward digital health. Training programs, institutional support, and user-friendly technology design may ensure that health care personnel feel both confident and motivated to use digital health technologies in home-based PPC [17,37,42].

Furthermore, the reported limited use of digital health technologies, combined with health care personnel’s requests for more digital health solutions, is noteworthy. These results may indicate that existing digital health technologies do not adequately meet their requirements for user-friendliness and seamless interoperability [10,14]. To address these challenges, engaging health care personnel in the needs assessment and design process of digital health can result in the creation of practical and valuable solutions that can enhance the quality of life for children with life-threatening and life-limiting conditions, as well as their families [4]. Additionally, to ensure seamless integration between different electronic health records and to avert connectivity issues for remote monitoring, it is crucial to establish the necessary infrastructure [4,12-14,17]. Ultimately, health care institutions should acknowledge the necessity of a robust infrastructure and an involving and supportive environment as their responsibility, rather than placing the burden on health care personnel in clinical practice [17,37].

The majority of health care personnel indicated that their last contact could not be replaced with technology and that digital health technologies should serve as a supplement rather than a replacement for in-person care. This hesitation to use digital health technologies instead of a physical presence is well documented in research on home-based PPC [4,6,7,12,14] and may be attributed to the unique nature of PPC, which addresses the physical, psychological, and spiritual needs of children and their families [1]. Additionally, this perception may partly reflect behavioral tendencies, as individuals often assume that established practices cannot be replaced until they experience alternative approaches [17]. Evidence suggests that attitudes toward real-time video consultation shift after direct exposure, indicating that initial resistance may not represent permanent barriers [35]. Similarly, the use of digital health technologies might affect health care personnel’s identities, values, and the perceived quality of patient care, as it may conflict with their commitment to providing holistic, compassionate, and personalized care [4,6,7]. This tension can lead to reluctance, as health care personnel may see digital health technologies as impersonal [5-7]. However, research shows that using health technology over time, or multiple in-person visits before introducing it, can help overcome doubt and foster familiarity and acceptance [36]. The gradual integration of digital health technologies may provide health care personnel with experiences that demonstrate how its use can align with their values, thereby supporting its implementation in practice. Additionally, health care personnel’s hesitation to use digital health technologies may be reduced by expanding their digital health care literacy beyond basic digital skills through targeted training programs [37,42]. Health care institutions should emphasize the importance of allowing sufficient time for health care personnel to adapt to new technologies and offer training that also focuses on developing social interaction, communication skills, self-reflection, and creativity [37,42]. Through this approach, digital health might support home-based PPC to be secure, collaborative, and effective.

### Strengths and Limitations

The major limitation of this study is the use of convenience sampling, which is associated with a lack of representativeness and potential selection bias [38]. Additionally, the relatively small sample size limits the generalizability of the results [38]. These factors should be considered when interpreting the results.

The distribution of the survey through email and social media raises concerns about potential bias, favoring those with digital access and higher digital health care literacy. This could skew the data from respondents and fail to represent non-respondents [26]. Furthermore, the survey did not capture the extent to which respondents were involved in providing home-based PPC. Participants were eligible if they provided care to children with life-threatening and life-limiting conditions in home, school, or respite settings, which may include varying levels of involvement. This variability could influence responses and should be considered when interpreting the results.

A strength of this study is the inclusion of different clinical health care personnel, including nurses, physicians, physiotherapists, and occupational therapists, from both primary and specialized services, as well as participants from various geographical regions across Norway. Thus, this study offers an overview of the nationwide use of digital health technologies among health care personnel providing home-based PPC. This study stands out as one of the first to gather responses not only from nurses and physicians [17] but also from physiotherapists and occupational therapists, who play essential roles in the interdisciplinary care team [1]. Regarding participation, 338 visits to the first page with information about this study, 198 people consented to participate, and 148 questionnaires were completed. The completion rate, defined as the ratio of individuals who submitted the final questionnaire page to those who agreed to participate [26], was about 75%. This indicates engagement and a willingness to participate among those who consented.

The focus of this study was placed on reporting the applicability for health care personnel providing home-based PPC rather than generalizability [38]. However, the eligibility criteria of the target population, combined with the description of the sample, should provide the reader with a rich understanding of the respondents and context, helping to discern whether the results would be applicable in their practice. The predominance of female participants reflects the gender distribution among health care personnel in Norway [43]. While this limits the ability to explore gender-related differences, it does not necessarily indicate a sampling bias, given the professional context.

This survey included a set of validated and/or previously used items and instruments, which is a strength of our work. We avoided overlap by removing items from the Norwegian Healthcare Personnel Survey on eHealth 2022 in favor of items from ITASH. However, the scale measuring digital health care literacy was translated into Norwegian specifically for this study and has not been used in Norway or among health care personnel before. It serves as an initial attempt to explore digital health care literacy alongside attitudes toward digital health in home-based PPC. Future research is needed to fully explore these phenomena.

### Conclusions

This study explored the use of digital health technologies by Norwegian health care personnel in home-based PPC and the association between their digital health care literacy and attitudes toward digital health. Despite high digital health care literacy and positive attitudes, the results indicated limited use of digital health technologies. This suggests that inadequate digital health solutions may create obstacles to their effective use in home-based PPC. Addressing these barriers is critical to enhancing the use of digital health technologies in home-based PPC.

Future research should explore how health care institutions can better integrate digital health technologies into their infrastructure and workflows. Additionally, research should examine behavioral factors that shape health care personnel’s perceptions, particularly how their attitudes evolve after direct experience with digital health technologies. Research can also explore how social interaction, communication, and personalized care can be effectively applied in combination with digital health. The goal is to ensure that home-based PPC supported by digital health does not compromise the quality of care provided to children and their families.

## Abbreviations

CHERRIES: Checklist for Reporting Results of Internet E-Surveys
CHIP: Children in Palliative Care
DHLS: Digital Health Care Literacy Scale
ITASH: Information Technology Attitude Scales for Health
PPC: Pediatric palliative care
STROBE: Strengthening the Reporting of Observational Studies in Epidemiology
